# Metastable Kitaev Magnets

**DOI:** 10.3390/molecules27030871

**Published:** 2022-01-27

**Authors:** Faranak Bahrami, Mykola Abramchuk, Oleg Lebedev, Fazel Tafti

**Affiliations:** 1 Department of Physics, Boston College, Chestnut Hill, MA 02467, USA; bahrami@bc.edu (F.B.); abramchukns@gmail.com (M.A.); 2 Laboratoire CRISMAT, ENSICAEN-CNRS UMR6508, 14050 Caen, France; oleg.lebedev@ensicaen.fr

**Keywords:** metastable, magnetism, topochemical

## Abstract

Nearly two decades ago, Alexei Kitaev proposed a model for spin-1/2 particles with bond-directional interactions on a two-dimensional honeycomb lattice which had the potential to host a quantum spin-liquid ground state. This work initiated numerous investigations to design and synthesize materials that would physically realize the Kitaev Hamiltonian. The first generation of such materials, such as Na${}_{2}$IrO${}_{3}$, α-Li${}_{2}$IrO${}_{3}$, and α-RuCl${}_{3}$, revealed the presence of non-Kitaev interactions such as the Heisenberg and off-diagonal exchange. Both physical pressure and chemical doping were used to tune the relative strength of the Kitaev and competing interactions; however, little progress was made towards achieving a purely Kitaev system. Here, we review the recent breakthrough in modifying Kitaev magnets via topochemical methods that has led to the second generation of Kitaev materials. We show how structural modifications due to the topotactic exchange reactions can alter the magnetic interactions in favor of a quantum spin-liquid phase.

## 1. Introduction

Recently, 4d/5d honeycomb layered materials have been vigorously studied due to their potential in realizing a quantum spin-liquid (QSL) ground state [1,2,3,4,5,6,7,8]. First introduced by Alexei Kitaev in 2006, the Kitaev model is an exactly solvable theoretical model with bond-dependent Ising interactions among spin-1/2 degrees of freedom on a two-dimensional (2D) honeycomb lattice, which is described by the Kitaev Hamiltonian: $H=-\sum K_{\gamma }S_{i}^{\gamma }S_{j}^{\gamma }$ [9]. The ground state of this system is magnetically frustrated and is predicted to be a QSL [9]. The applications of a Kitaev QSL in quantum information and the possibility of realizing Majorana fermions have inspired numerous investigations into quasi-2D honeycomb materials [1,3,10,11,12]. Such materials are colloquially labelled Kitaev magnets as they support a sizable Kitaev interaction; however, one needs to consider that other interactions such as Heisenberg exchange are also present and compete with the Kitaev interaction in the so-called Kitaev magnets [3,10].

**Figure 1:**
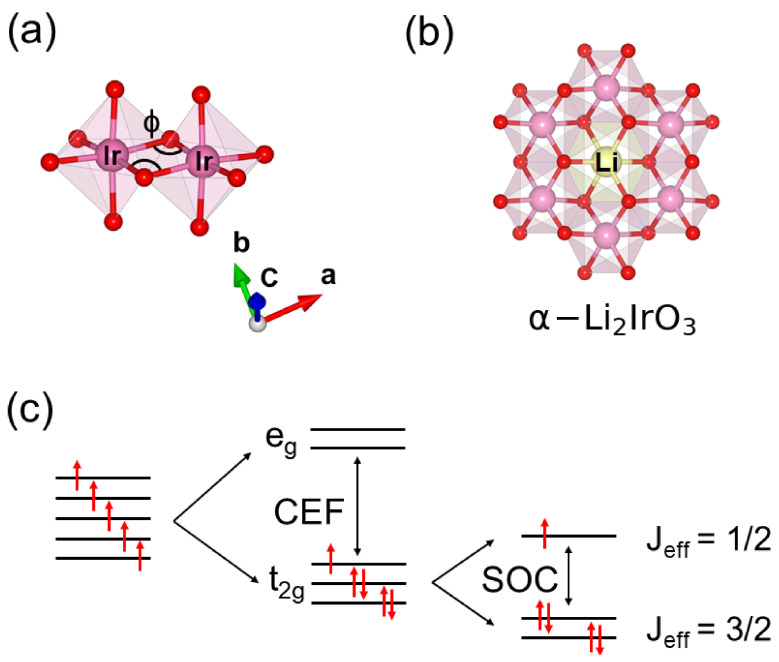
(**a**) The bond angle (ϕ) between edge-shared octahedral units plays a significant role in tuning the magnetic interactions. (**b**) Edge-sharing octahedral units create a honeycomb structure in Kitaev magnets such as α-Li${}_{2}$IrO${}_{3}$ and Na${}_{2}$IrO${}_{3}$. (**c**) Interplay between CEF and SOC creates the isospin-1/2 state in the Kitaev magnets.

The first generation of Kitaev magnets, namely Na${}_{2}$IrO${}_{3}$, α-Li${}_{2}$IrO${}_{3}$, Li${}_{2}$ RhO ${}_{3}$, and α-RuCl${}_{3}$, were synthesized using conventional solid-state methods at high temperatures (*T* > 700 °C). In these materials, heavy transition metal ions (Ru${}^{3+}$, Rh${}^{4+}$, and Ir${}^{4+}$) are octahedrally coordinated with oxygen or chlorine atoms (Figure 1a), and the edge-sharing octahedra create honeycomb layers (Figure 1b). The combination of octahedral crystal electric field (CEF) and strong spin-orbit coupling (SOC) splits the five-fold degenerate *d*-levels and leaves one electron in the isospin-1/2 ($J_{\mathrm{eff}}$ = 1/2) state necessary for the Kitaev model (Figure 1c) [1,4,7,9,13].

Finding new Kitaev magnets, beyond the first-generation compounds, has become a frontier challenge in solid-state chemistry. Prior attempts to replace Na with K in Na${}_{2}$IrO${}_{3}$ or replacing Cl with Br in α-RuCl${}_{3}$ has led to other stable phases with different structures instead of the honeycomb lattice [14,15]. The amount of physical pressure required to substantially tune the interactions is too high [16] and chemical doping leads to a change of spin state [17]. Therefore, recent success in synthesizing a second generation of Kitaev magnets where magnetic interactions can be tuned by topochemical methods has revitalized the field. In this review, we will first explain the different types of exchange reactions (partial and complete), then discuss the interplay between topochemical reactions and magnetism, and finally present heat capacity and magnetization data to compare the properties of the first- and second-generation Kitaev magnets.

## 2. Topotactic Exchange Reactions

**Figure 2:**
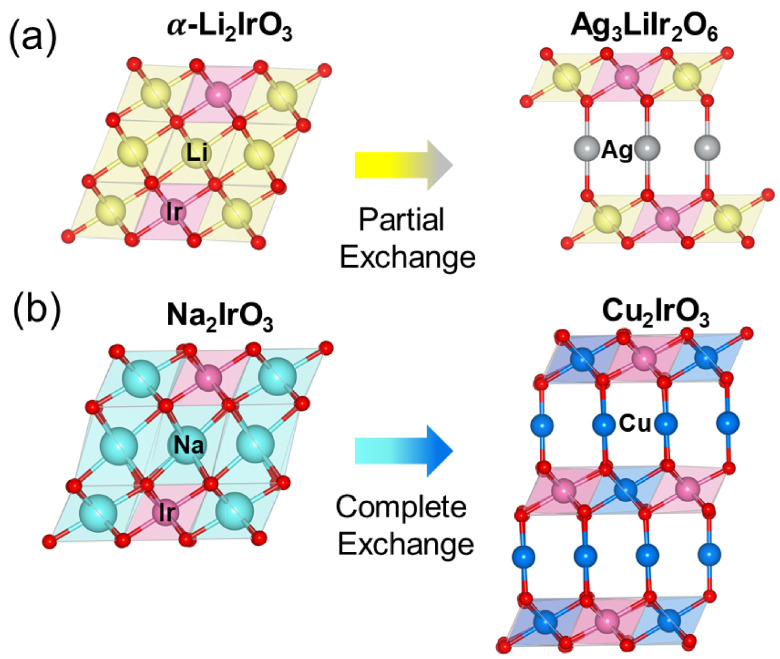
Synthesis of the second-generation Kitaev magnets from the first-generation materials through (**a**) partial and (**b**) complete exchange reactions. Both generations have honeycomb layers. The topochemical change of interlayer coordination from octahedral to linear modifies the intra-layer Ir-O-Ir bond angles due to the change of oxygen positions.

The second-generation Kitaev magnets are metastable compounds, i.e., they have a higher enthalpy of formation and a lower decomposition threshold compared to stable counterparts [18]. Thus, it is impossible to synthesize them with conventional solid-state methods at high temperatures. Instead, they are stabilized through topochemical reactions from the first-generation compounds under mild conditions. As shown schematically in Figure 2 the global symmetries of the unit cell do not change during a topochemical reaction. However, the local parameters such as bond lengths and bond angles are modified efficiently.

Topotactic exchange reactions can be either partial (Figure 2a) or complete (Figure 2b). The most general formulation of a partial exchange reaction is

(1) $$2A_{2}{\mathrm{MO}}_{3}+3\mathrm{BX}\;\to \;B_{3}{\mathrm{AM}}_{2}O_{6}+3\mathrm{AX}$$

where the interlayer A-atoms (typically Li or Na) in a stable honeycomb structure A${}_{2}$MO${}_{3}$ are exchanged with the B-atoms (typically Cu, Ag, and H) from a halide, nitrate, or sulfate compound BX. For example, Figure 2a corresponds to A = Li, B = Ag, M = Ir, and X = NO${}_{3}$ for the synthesis of Ag${}_{3}$LiIr${}_{2}$O${}_{6}$ from α-Li${}_{2}$IrO${}_{3}$. Replacing the interlayer Li atoms by H, Cu, or Ag, in α-Li${}_{2}$IrO${}_{3}$ has recently produced H${}_{3}$LiIr${}_{2}$O${}_{6}$, Cu${}_{3}$LiIr${}_{2}$O${}_{6}$, and Ag${}_{3}$LiIr${}_{2}$O${}_{6}$, respectively [19,20,21,22,23].

In a complete topotactic exchange reaction, all A-atoms within and between the layers are replaced by the B-atoms.

(2) $$A_{2}{\mathrm{MO}}_{3}+2\mathrm{BX}\;\to \;B_{2}{\mathrm{MO}}_{3}+2\mathrm{AX}$$

For example, Figure 2b corresponds to A = Na, B = Cu, M = Ir, and X = Cl for the synthesis of Cu${}_{2}$IrO${}_{3}$ from Na${}_{2}$IrO${}_{3}$. A complete exchange reaction is much less likely to happen and so far, Cu${}_{2}$IrO${}_{3}$ is the only known system in this category [24]. It is noteworthy that the copper atoms in Cu${}_{2}$IrO${}_{3}$ are not entirely in a Cu${}^{+}$ state. Both X-ray absorption and electron energy loss spectroscopy (XAS and EELS) confirmed a mixed valence of Cu${}^{+}$/Cu${}^{2+}=1/1$ within the honeycomb layers [25]. A mixed valence of copper induces a mixed valence of iridium (Ir${}^{+3}$/Ir${}^{+4}$) and leads to magnetic disorder and spin-glass behavior [25,26].

Topochemical reactions enable us to considerably change the coordination environment, bond lengths, and bond angles between the first- and second-generation compounds (Figure 2). In a partial exchange reaction, the octahedrally coordinated interlayer alkaline atoms (A) in the parent compounds such as $A_{2}{\mathrm{BO}}_{3}$ are replaced by B = H, Cu, or Ag atoms with a linear (dumbbell) coordination in the second-generation compound $B_{3}{\mathrm{AM}}_{2}O_{6}$ (Figure 2a). This change in the interlayer chemical bond coordination increases the interlayer spacing when B = Cu or Ag, and decreases it when B = H. It also changes the position of O atoms in the honeycomb layers of the product (second-generation) compounds and thus changes the bond angles and bond lengths that determine the magnetic interactions. In a complete exchange reaction (Figure 2b), not only are the interlayer alkaline atoms (A) and their associated chemical bonds changed, but the alkaline cations within the honeycomb layers are also replaced by the B-atoms. The increasing of the interlayer spacing and weakening of interlayer chemical bonds are still valid in a complete exchange reaction.

## 3. Synthesis Details

**Figure 3:**
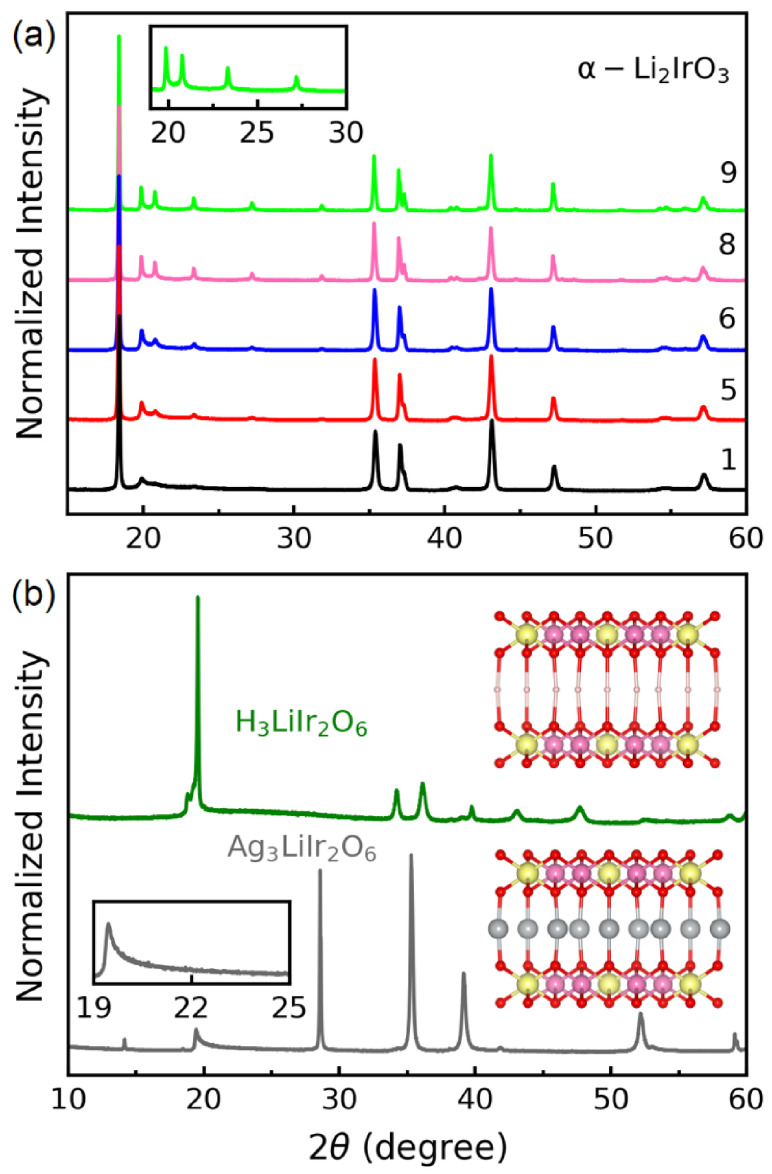
(**a**) After each heat cycle, the powder X-ray pattern of α-Li${}_{2}$IrO${}_{3}$ shows more pronounced peaks, especially between 20 and 30 degrees where the honeycomb Bragg peaks appear. The number of times each sample has been reheated is shown on the right above its respective pattern. (**b**) The X-ray patterns of two second-generation Kitaev magnets, H${}_{3}$LiIr${}_{2}$O${}_{6}$ (green) and Ag${}_{3}$LiIr${}_{2}$O${}_{6}$ (gray data, reproduced from [28]). The inset shows the asymmetric broadening of the honeycomb Bragg peaks in Ag${}_{3}$LiIr${}_{2}$O${}_{6}$ due to stacking faults. In H${}_{3}$LiIr${}_{2}$O${}_{6}$, the honeycomb peaks are hardly discernible due to high structural disorder.

The first-generation Kitaev magnets are prepared via conventional solid-state reaction at high temperatures (T ≥700 K) in air, under vacuum, or under the flow of oxygen/argon gas [7,13,27]. To improve the sample quality and remove stacking faults, it is necessary to perform successive stages of grinding and heating. For example, the X-ray patterns in Figure 3a show that the quality of α-Li${}_{2}$IrO${}_{3}$ samples improve by repeating the heat cycles. Specifically, the superstructure peaks between 20 and 30 degrees (inset of Figure 3a) that represent the honeycomb ordering become more pronounced in each iteration. Typically, improving the quality of the first-generation compound will improve the quality of the second-generation material after the exchange reaction [28].

The topotactic cation exchange reaction must be conducted at low temperatures (T≤400 K) [24,28,29], since higher temperatures will decompose the metastable product. The second-generation Kitaev magnets are prepared by modifying the interlayer atoms and the associated chemical bonds, and therefore they have more stacking faults than their parent compounds [28,30]. This can be seen in the inset of Figure 3b that shows an asymmetric broadening of the honeycomb Bragg peaks in Ag${}_{3}$LiIr${}_{2}$O${}_{6}$. Unlike solid-state reactions, topotactic exchange cannot be repeated to improve the sample quality. Thus, removing the stacking faults in these materials remains an open challenge.

Details of the synthesis procedures for Cu${}_{2}$IrO${}_{3}$ and Ag${}_{3}$LiIr${}_{2}$O${}_{6}$ have been published by Abramchuk and Bahrami et al. previously [19,24,28]. Here, we present more details about the synthesis of H${}_{3}$LiIr${}_{2}$O${}_{6}$ based on the earlier work of Bette et al. [31]. Polycrystalline samples of H${}_{3}$LiIr${}_{2}$O${}_{6}$ are synthesized using a modified version of Equation (1).

(3) $$4{\mathrm{Li}}_{2}{\mathrm{IrO}}_{3}+3H_{2}{\mathrm{SO}}_{4}\;\to \;2H_{3}{\mathrm{LiIr}}_{2}O_{6}+3{\mathrm{Li}}_{2}{\mathrm{SO}}_{4}$$

After synthesizing a high-quality sample of α-Li${}_{2}$IrO${}_{3}$ (Figure 3a), approximately 300 mg of the material was added to a 10 mL Teflon-lined steel autoclave filled with H${}_{2}$SO${}_{4}$ acid (1 M solution) and heated to 120 °C for several days. After completing the reaction, the product was washed with water and the quality was verified using X-ray diffraction (Figure 3b).

## 4. Stacking Faults

A comparison between the insets of Figure 3a,b suggests fewer stacking faults in α-Li${}_{2}$IrO${}_{3}$ (sharp and well-separated Bragg peaks from the honeycomb layers) and considerable stacking faults in Ag${}_{3}$LiIr${}_{2}$O${}_{6}$ (broadened peaks). The asymmetric broadening of honeycomb peaks is known as the Warren line shape, which is a signature of stacking disorder [32]. The higher amount of stacking faults in the second-generation Kitaev magnets is due to the interlayer chemistry. As shown in Figure 2, each interlayer Li atom in α-Li${}_{2}$IrO${}_{3}$ is octahedrally coordinated with three oxygen atoms from the top and three from the bottom honeycomb layers. In contrast, each Ag atom in Ag${}_{3}$LiIr${}_{2}$O${}_{6}$ is connected to only one O atom from the top and one from the bottom layer in a dumbbell (linear) coordination. The weak dumbbell bonds are responsible for the larger interlayer separation in Ag${}_{3}$LiIr${}_{2}$O${}_{6}$ and the presence of more stacking faults compared to α-Li${}_{2}$IrO${}_{3}$ [33].

**Figure 4:**
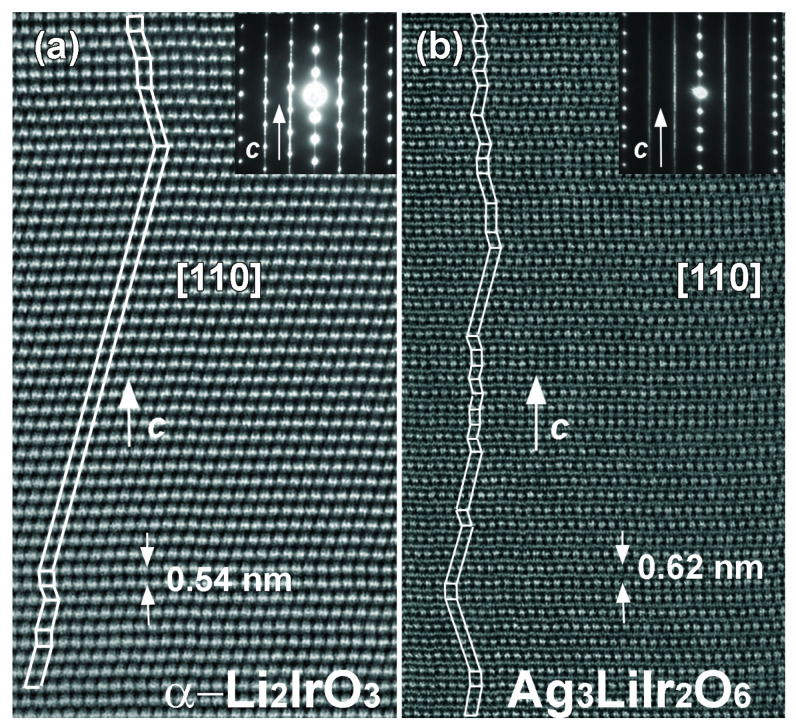
HAADF-TEM images from (**a**) α-Li${}_{2}$IrO${}_{3}$ and (**b**) Ag${}_{3}$LiIr${}_{2}$O${}_{6}$. The images show an abundance of stacking faults in Ag${}_{3}$LiIr${}_{2}$O${}_{6}$ unlike α-Li${}_{2}$IrO${}_{3}$, due to the weaker interlayer bonding in the former. The electron diffraction patterns are presented as insets and reveal less streaking in α-Li${}_{2}$IrO${}_{3}$ due to fewer stacking faults compared to Ag${}_{3}$LiIr${}_{2}$O${}_{6}$.

Direct lattice imaging with transmission electron microscopy (TEM) is a powerful tool to study the stacking faults. Figure 4a,b (reproduced from Ref. [28]) are high angle annular dark-field TEM (HAADF-TEM) images of α-Li${}_{2}$IrO${}_{3}$ and Ag${}_{3}$LiIr${}_{2}$O${}_{6}$ samples, respectively. Although the stacking sequence in α-Li${}_{2}$IrO${}_{3}$ can be flawless for up to 50 unit cells, Ag${}_{3}$LiIr${}_{2}$O${}_{6}$ shows a maximum of 5 unit cells stacked without faults (in the form of twisting between the layers). In H${}_{3}$LiIr${}_{2}$O${}_{6}$, the small size of H atoms and their high mobility make the chemical bonds even weaker than in Ag${}_{3}$LiIr${}_{2}$O${}_{6}$. As such, H${}_{3}$LiIr${}_{2}$O${}_{6}$ has the highest degree of stacking faults among the second-generation Kitaev magnets [29,30,31]. Therefore, the honeycomb peaks of H${}_{3}$LiIr${}_{2}$O${}_{6}$ are not resolved by X-rays (Figure 3b).

## 5. Tuning Magnetic Interactions with Topochemical Methods

As shown in Figure 2, the monoclinic unit cell and the honeycomb ordering in the 2D layers remain unchanged before and after exchange reactions. However, the change of interlayer coordination from octahedral to dumbbell modifies the M-O-M bond angles within the honeycomb layers (Figure 1a and Figure 2). Superexchange magnetic interactions are sensitive to a change of bond angles and thus, topochemical reactions can be used to tune the magnetic interactions. There are at least three terms in the magnetic Hamiltonian of the Kitaev materials.

(4) $$H=\sum_{\langle i,j\rangle \in \alpha \beta (\gamma )}\left[-K_{\gamma }S_{i}^{\gamma }S_{j}^{\gamma }+JS_{i}\cdot S_{j}+\Gamma \left(S_{i}^{\alpha }S_{j}^{\beta }+S_{i}^{\beta }S_{j}^{\alpha }\right)\right]$$

The Kitaev term (*K*) favors QSL, the Heisenberg term (*J*) favors antiferromagnetic (AFM) ordering, and the off-diagonal exchange term (Γ) controls details of the ordered structure. All three terms can be modified via topochemical reactions as follows.

**Figure 5:**
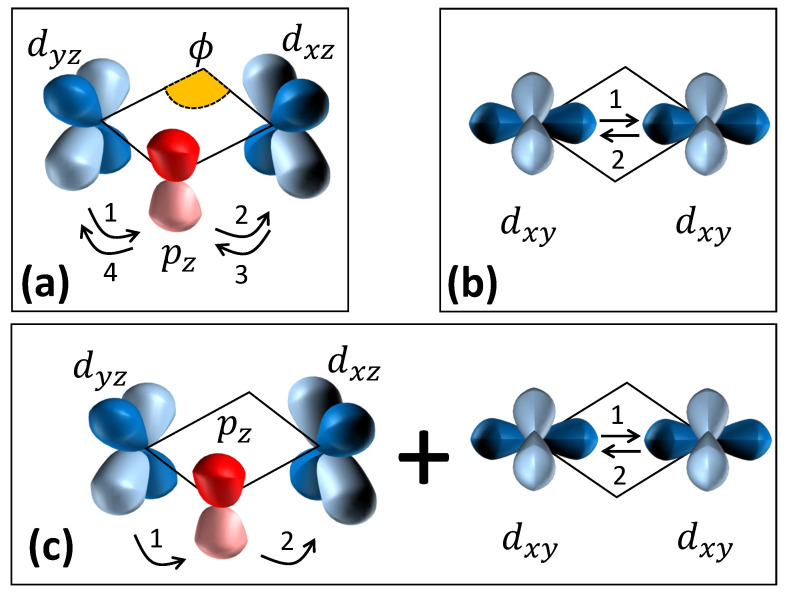
Exchange paths for (**a**) *K*, (**b**) *J*, and (**c**) Γ terms in Equation (4). The *d* and *p* orbitals are painted in blue and red, respectively. The numbers show the hopping sequence in the perturbation.

Figure 5 shows the individual exchange paths for each term in Equation (4). The Kitaev term is an indirect exchange interaction with hopping matrix elements $t_{dpd}$ between the $d_{xz}$, $p_{z}$, and $d_{yz}$ orbitals (Figure 5a) [34,35]. In addition to the indirect exchange (*K*), Figure 5b shows a direct exchange path for the Heisenberg interaction (*J*) with hopping matrix element $t_{dd}$ between $d_{xy}$ orbitals, leading to $J∼t_{dd}^{2}/U$ in Equation (4) [36]. Finally, a combination of direct and indirect paths in Figure 5c leads to the symmetric off-diagonal exchange, $\Gamma ∼t_{dpd}t_{dd}J_{H}/U^{2}$, where $J_{H}$ is the Hund’s coupling between the $e_{g}$ and $t_{2g}$ orbitals [37,38]. The hopping matrix elements ($t_{dd}$ and $t_{dpd}$) are tuned by the M-O-M bond angle and the M-M distance which can be tuned by the exchange reactions. For example, (i) the change of oxygen positions within the honeycomb layers due to the change of interlayer coordination in Figure 2 modifies the M-O-M bond angle (ϕ in Figure 1a and Figure 5a) and therefore tunes $t_{dpd}$; (ii) according to theoretical calculations [1], the Heisenberg interaction is canceled between the opposite paths if the bond angle ϕ is close to $90^{\circ }$ (Figure 1a and Figure 5a); (iii) the hybridization between the Ag *d*-orbitals between the layers and O *p*-orbitals within the layers tunes the ratio of $t_{dpd}/t_{dd}$.

## 6. Magnetic Characterization of Metastable Kitaev Materials

**Figure 6:**
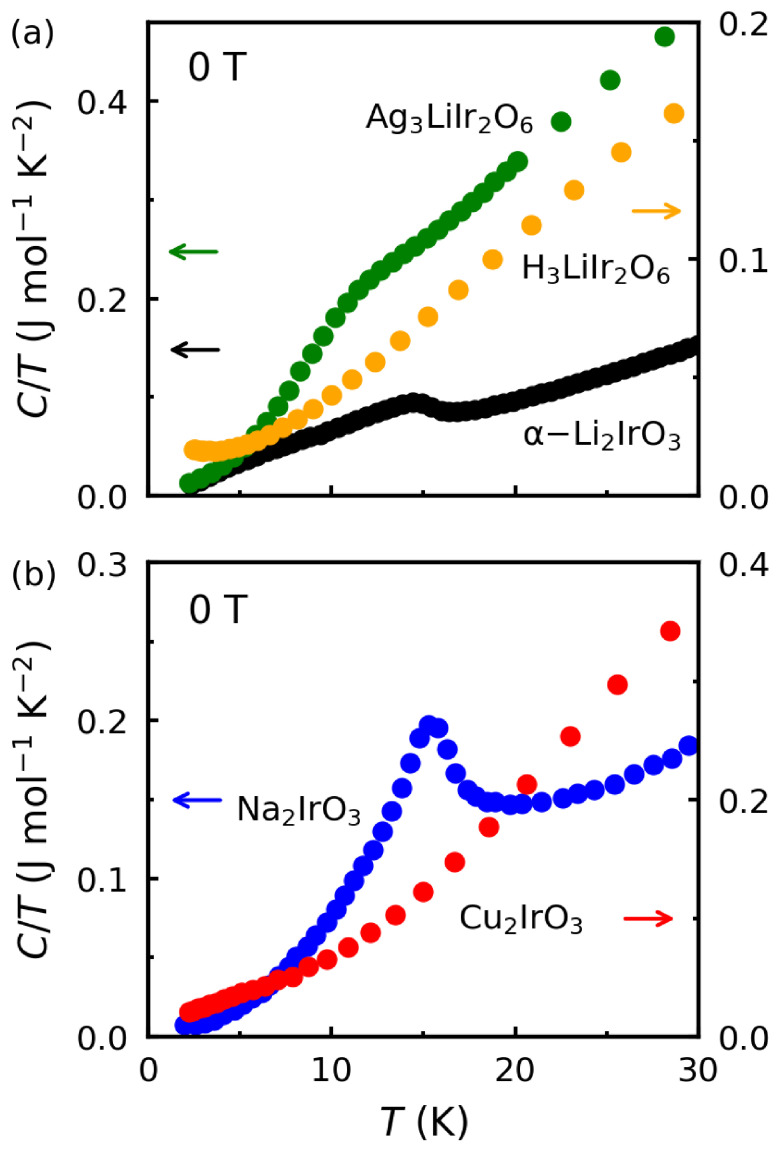
(**a**) Heat capacity (C/T) plotted as a function of temperature below 30 K for the first-generation Kitaev magnet α-Li${}_{2}$IrO${}_{3}$ and its second-generation derivatives Ag${}_{3}$LiIr${}_{2}$O${}_{6}$ and H${}_{3}$LiIr${}_{2}$O${}_{6}$. The data for α-Li${}_{2}$IrO${}_{3}$ and Ag${}_{3}$LiIr${}_{2}$O${}_{6}$ are reproduced from Refs. [2,28]. (**b**) A similar comparison is made between Na${}_{2}$IrO${}_{3}$ (first generation) and Cu${}_{2}$IrO${}_{3}$ (second generation). The data are reproduced from Ref. [24].

To demonstrate the effect of topochemical modifications on the magnetic interactions (Equation (4) and Figure 5), we compare the heat capacity and magnetic susceptibility of the first- and second-generation Kitaev magnets. The peak in the heat capacity of α-Li${}_{2}$IrO${}_{3}$ in Figure 6a confirms long-range magnetic ordering at $T_{N}=15$ K. The order has been characterized as an incommensurate spiral by recent neutron scattering and muon spin relaxation (μSR) experiments [5,8]. As shown in Figure 6a, this peak is shifted to lower temperatures in Ag${}_{3}$LiIr${}_{2}$O${}_{6}$ and seemingly disappeared in H${}_{3}$LiIr${}_{2}$O${}_{6}$. The suppression of $T_{N}$ in second-generation compounds Ag${}_{3}$LiIr${}_{2}$O${}_{6}$ and H${}_{3}$LiIr${}_{2}$O${}_{6}$ is a positive sign of approaching the QSL phase, where long-range order is replaced by long-range quantum entanglement [3,10]. A recent μSR experiment [28] has shown a similar incommensurate spiral order in Ag${}_{3}$LiIr${}_{2}$O${}_{6}$; however, the long-range order develops at 8 K in Ag${}_{3}$LiIr${}_{2}$O${}_{6}$, well below $T_{N}=15$ K in α-Li${}_{2}$IrO${}_{3}$. Thus, the topochemical modification of bond angles seems to strengthen *K* and weaken *J* in Equation (4). A recent nuclear magnetic resonance (NMR) experiment has shown the absence of long-range order in H${}_{3}$LiIr${}_{2}$O${}_{6}$, which is another promising result toward the discovery of a QSL phase [29].

A similar trend is observed in Figure 6b for the first-generation material Na${}_{2}$IrO${}_{3}$ that shows a peak at $T_{N}=15$ K and its second-generation counterpart Cu${}_{2}$IrO${}_{3}$ that does not show a peak but seems to have a broad anomaly below 5 K. Neutron scattering experiments have confirmed a zigzag AFM order in Na${}_{2}$IrO${}_{3}$ [39]. Recent μSR and NMR experiments have revealed a coexistence of static and dynamic magnetism below 5 K in Cu${}_{2}$IrO${}_{3}$ but without a long-range order, suggesting proximity to the QSL phase [25,40].

**Figure 7:**
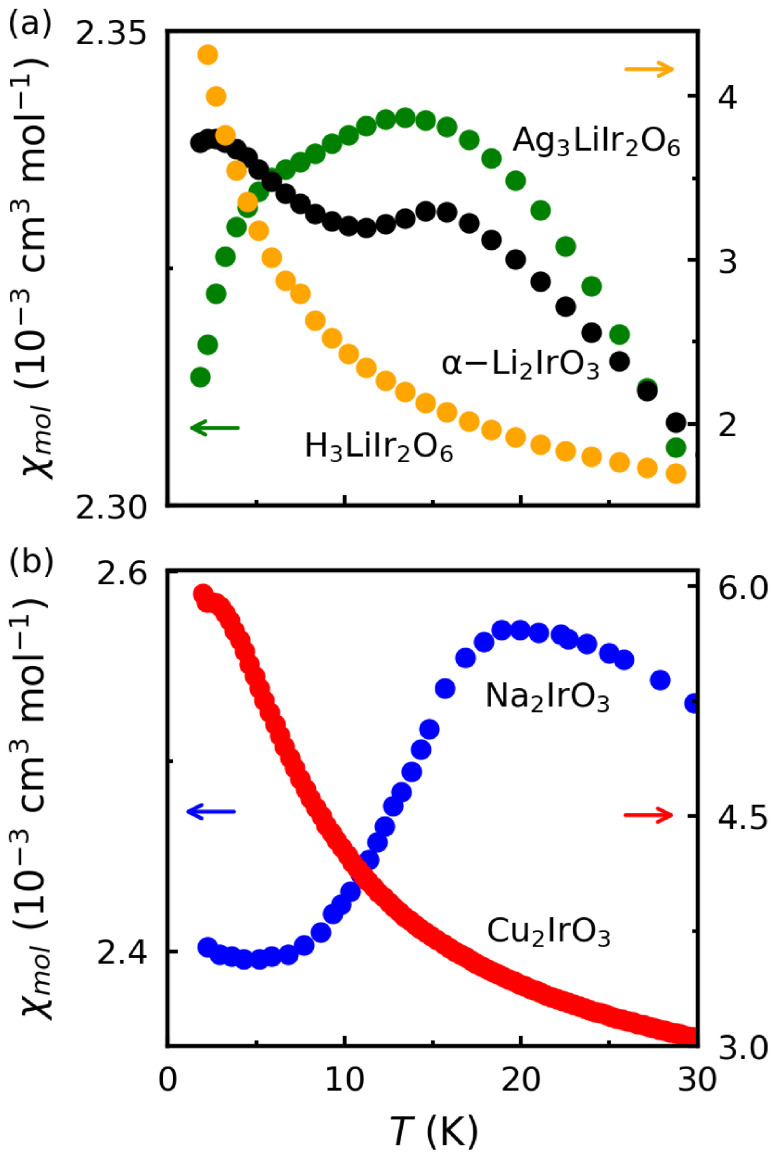
(**a**) Magnetic susceptibility (χ) plotted as a function of temperature below 30 K for the first-generation Kitaev magnet α-Li${}_{2}$IrO${}_{3}$ and its second-generation derivatives Ag${}_{3}$LiIr${}_{2}$O${}_{6}$ and H${}_{3}$LiIr${}_{2}$O${}_{6}$. The data for α-Li${}_{2}$IrO${}_{3}$ and Ag${}_{3}$LiIr${}_{2}$O${}_{6}$ are reproduced from Refs. [19,28] (The y range for α-Li${}_{2}$IrO${}_{3}$ is from 4.8 to 5.3). (**b**) A similar comparison is made between Na${}_{2}$IrO${}_{3}$ (first generation) and Cu${}_{2}$IrO${}_{3}$ (second generation). The data for Na${}_{2}$IrO${}_{3}$ and Cu${}_{2}$IrO${}_{3}$ are reproduced from Refs. [2,24].

The suppression of magnetic ordering due to topochemcial changes in metastable Kitaev magnets is also observed in the magnetic susceptibility data. Figure 7a shows the magnetic susceptibility of α-Li${}_{2}$IrO${}_{3}$ (black curve) with a clear anomaly at $T_{N}=15$ K indicating the incommensurate spiral AFM order. The green curve representing Ag${}_{3}$LiIr${}_{2}$O${}_{6}$ shows two downturns at $T_{F}=14$ K and $T_{N}=8$ K, corresponding to the onsets of short-range and long-range magnetic orders, respectively [28]. The orange curve representing H${}_{3}$LiIr${}_{2}$O${}_{6}$ does not show any evidence of magnetic ordering. Figure 7b shows a similar trend, where the first-generation material Na${}_{2}$IrO${}_{3}$ orders at $T_{N}=15$ K and the second-generation material Cu${}_{2}$IrO${}_{3}$ shows a small peak at 2 K, evidence of short-range spin freezing instead of long-range order.

## 7. Challenges and Opportunities

The above results are exciting; however, they need to be interpreted with caution. Topotactic exchange reactions increase disorder that has adverse effects on magnetism. A recent TEM study has shown that the silver atoms in Ag${}_{3}$LiIr${}_{2}$O${}_{6}$ can enter the honeycomb layers and form small inclusions (up to 50 atoms) that disrupt the magnetic ordering [28]. Such a structural disorder can spuriously hide the long-range order and be misinterpreted as evidence of a QSL phase. As noted earlier, H${}_{3}$LiIr${}_{2}$O${}_{6}$ is even more disordered compared to Ag${}_{3}$LiIr${}_{2}$O${}_{6}$ due to the high mobility of the H atoms, which causes bond randomness and site vacancies within the honeycomb layers [31]. Recent theoretical works show that the absence of magnetic ordering in H${}_{3}$LiIr${}_{2}$O${}_{6}$ may be due to bond randomness and a large number of vacancies [41,42]. Thus, the most important challenge in this field is to optimize the synthesis conditions for a minimum amount of disorder and to find methods of annealing away the stacking faults and vacancies.

One promising approach to minimize the structural disorder in the second-generation Kitaev magnets is to provide single-crystal specimens of this family. Single crystals will also enable accurate determination of the interlayer and intra-layer exchange couplings. Both Raman and nuclear magnetic resonance (NMR) experiments can provide information about the fractionalized (Majorana) excitations in single crystals [23,43].

Metastable Kitaev magnets have opened a new window of opportunity to realizing the quantum spin-liquid ground state. The Majorana excitations of such materials will form the building blocks of a solid-state quantum computer [44]. Braiding algorithms and logical gates have been theoretically developed for such computers [45]. It remains an open challenge for the solid-state chemistry community to synthesize the appropriate materials for such models. Another intriguing opportunity is to find unconventional superconductivity in the Kitaev magnets [46], an exciting theoretical prediction that awaits experimental discovery.

## Data Availability

All data are available from the authors upon request.
